# A minimal descriptor of an ancestral recombinations graph

**DOI:** 10.1186/1471-2105-12-S1-S6

**Published:** 2011-02-15

**Authors:** Laxmi Parida, Pier Francesco Palamara, Asif Javed

**Affiliations:** 1 Computational Genomics, IBM T J Watson Research, Yorktown, New York, USA; 2 Columbia University, New York, USA; 3 The work done during an internship at IBM T J Watson Research Center

## Abstract

**Background:**

Ancestral Recombinations Graph (ARG) is a phylogenetic structure that encodes both duplication events, such as mutations, as well as genetic exchange events, such as recombinations: this captures the (genetic) dynamics of a population evolving over generations.

**Results:**

In this paper, we identify structure-preserving and samples-preserving core of an ARG *G* and call it the minimal descriptor ARG of *G*. Its structure-preserving characteristic ensures that all the branch lengths of the marginal trees of the minimal descriptor ARG are identical to that of *G* and the samples-preserving property asserts that the patterns of genetic variation in the samples of the minimal descriptor ARG are exactly the same as that of *G*. We also prove that even an unbounded *G* has a finite minimal descriptor, that continues to preserve certain (graph-theoretic) properties of *G* and for an appropriate class of ARGs, our estimate (Eqn 8) as well as empirical observation is that the expected reduction in the number of vertices is exponential.

**Conclusions:**

Based on the definition of this lossless and bounded structure, we derive local properties of the vertices of a minimal descriptor ARG, which lend itself very naturally to the design of efficient sampling algorithms. We further show that a class of minimal descriptors, that of binary ARGs, models the standard coalescent exactly (Thm 6).

## Background

The study of genetic evolution of populations is an important problem and myriad aspects of this have been studied extensively for the past few decades. This problem is regaining momentum as more and more detailed genomes of different organisms, highlighting the unexpected diversity within a species, become available [1]. There are two broad directions to studying and understanding this diversity. One is through model-based population simulation studies: this helps hypothesize various evolutionary constraints and conditions and understand the observed population structures in that context. While it is impossible to model every detail of all the genetic events, very good statistical processes summarizing the series of genetic events exist [2-4]. Moreover, model based methods have been at the heart of various demographics-aware approaches [5-7]. The second direction is to reconstruct a plausible evolutionary history given the observed population structure as extant samples of chromosomes. In the context of human data, the reconstruction of trees from genomic data under uni-linear transmission, such as nonrecombining Y chromosome (NRY) or mitochondrial data is well accepted [8]. However, evolutionary reconstruction of recombining portions of the genome continues to be a challenge. Under these conditions, the second direction is arguably hard and reconstruction methods [9,10] have been evaluated in various orchestrated evolution scenarios [11]. In this paper, we introduce a minimal descriptor that plays a critical role in both the directions of study. Firstly, it does not compromise any detail of the genetic dynamics for simulation studies and secondly, leads itself to a structure amenable for reconstruction studies. Finally, since the minimal descriptor is very compact (as well as exact), it can form the basis for statistical inferencing methods as well.

The central mathematical object of study in this context is the Ancestral Recombinations Graph (ARG): the coalescent with recombination describes the genealogies underlying the common evolutionary history of samples of chromosomes from unrelated individuals of an ideal population [12,13]. This is also called the *standard coalescent model* in literature. This object has been studied intensively in literature primarily in the context of simulations. In [14], the more general version of the ARG was studied as a random graph to address the general reconstructability question. In this paper, the attempt is to understand some inherent characteristics of the ARG from a reconstruction perspective. Not surprisingly, this has implications in simulations as well.

In the context of simulations, Hudson introduced *MS* the seminal implementation to sample sequences from a population evolving under the Wright Fisher model [15], It is important to point out a subtlety here. Usually under the coalescent model, the coalescence is between exactly two lineages and multiple genetic events do not occur in the same generation in the common evolutionary history. These simplifications help in defining the model as a ordered sequence of events as well as in estimating the time from one event to the next. In the software *GENOME*[16], instead of simulating the time to next event, the authors simulate the coalescent and recombination events at every generation proceeding backwards in time. Thus this models an evolutionary history, more general than the standard coalescent model. In the random graphs framework in [14], the *genetic exchange model* or *mixed subgraph* represented this more general model. In this paper, to avoid confusion in terminologies, we call such a general model simply the *generic* ARG and unless specified otherwise an ARG refers to a generic ARG. On the other hand we call the standard coalescent model as the *binary* ARG, for obvious reasons. While the above methods generate events backwards in time, an orthogonal approach, introduced in [17], samples the events along the sequence. This is called the *Spatial Algorithm* (SA) and one of its characteristic effects is that the density of recombination breakpoints increases as one moves along the sequence. Another (perhaps related) characteristic of SA is that the process is not Markovian. The *Sequentially Markov Coalescent*[18] introduces modifications to the process to make the structure Markovian. Based on this model, in *FastCoal*[19], the authors use an additional heuristic of retaining only a subset of local trees while moving along the sequence. However in all three, a probabilistic formulation of the underlying random mathematical object is not obvious. It turns out that even the Markovian structures only approximate the standard coalescent model. While each model is defined algorithmically as a sequence of precise steps, yet the reason for this lack of exactness is not clear enough to provide algorithmic modifications to close or reduce the gap with the standard model. On the other hand, the random-graphs framework [14] leads to a procedure-independent model, the *minimal descriptor of binary ARGs*, that is not only very compact but also exactly models the standard coalescent. Our ideas stem from a graph theoretic viewpoint of the generic ARG. We define *structure-preserving* and *samples-preserving* transforms of a generic *G* which maintain the invariance of the marginal genealogies and the samples (hence genetic variation patterns) respectively, called a *minimal descriptor*, that exactly models the generic ARG. This setting helps us in providing mathematical proofs of exactness of the model (Thms 2 and 6) as well as in deriving the other properties from the model (Thms 3-5). The local properties of the nodes lend themselves naturally to designing sampling algorithms for the method-independent model, say by modifications to Hudson’s algorithm (Results section).

### Background

As seen above, ARG forms the basis for most systematic simulation studies. It is important to point out another subtlety here. An ARG is a random object and there are many (infinite) *instances* of the ARG. Usually, when we say that a topological property holds for the ARG, we mean that the property that holds for every instance of the ARG, i.e. the property holds with probability 1. Note that some may hold for a subset of instances (such as unboundedness).

Focusing on the topology of the ARG, and its effect on the samples, provides us with insights to identify vertices that “do not matter”. Modeling these as missing nodes in the ARG, leads to a core that preserves the essential characterisitcs. We begin the exposition by recalling some important topological characteristics of the random ARG, which is defined by at least two parameters: *K*, the number of extant samples and 2*N*, the population size at a generation. A Grand Most Recent Common Ancestor (GMRCA), plays an important role in restricting the zone of interest in the common evolutionary structure. A GMRCA is defined as a unit whose genetic material is ancestral to all the genetic materials in all the extant samples [8]. Thus while the relevant common evolutionary history of some *K >* 1 units is potentially unbounded, it is reasonable to bound this structure of interest with this single GMRCA. Thus *when a GMRCA exists, it is unique* and and we say the ARG is *bounded*. When an ARG has no GMRCA, we call it *unbounded*.

The least common ancestor (LCA) of a set of vertices *V* in a graph is defined as a common ancestor of *V* with no other common ancestor of *V* on any path from the LCA to any vertex of *V*. In our earlier work [14], we presented a combinatorial treatment of the ARG based on random graphs. The (directed) graph representation is acyclic, a root is analogous to a GMRCA, and the leaf nodes to the extant samples. Though tantalizingly similar, GMRCA and LCA do not define the same entity in an ARG. The implications of this treatment relevant to this paper is summarized as follows.

The common evolutionary history of an ideal population can be modeled as a random graph. Due to the “ancestor without ancestry” paradox, the topology alone of this graph is not adequate and the nodes or edges must be embellished with additional information, which is the genetic material they transmit. Then

**Theorem 1 (Par10)***1. G satisfies the bounded-degree property.*

(In the random graph model, the ARG is defined on an infinite set of vertices. However, each vertex has not only finite, but a bounded number of incident edges, determined by the input parameters alone. Note that this is called the *bounded-degree* property, which is one of the measures of simplicity of a graph.)

*2. For every G on K >* 1 *extant samples, there exists some M ≥* 1*, such that G is the union of M overlapping trees (or forests), each with the same K extant samples. This is written as G =∪_T∈Τ_T, where Τ is the set of M trees.*

(Thus this *Genetic Exchange Model* gives an alternative parametrization of an ARG, with the number of non-mixing segments *M*)

3. In the embellished graph, the GMRCA is the Least Common Ancestor with Ancestry (LCAA) of all the K extant samples, where ‘ancestry’ is deduced from the embellishments, The topological definition of the GMRCA is as follows.: It is the LCA of the LCAs of the M embedded trees (of 2 above).

Based on (2) above, we set up the problem for studying the ARG and its structure-preserving core in the next section. Statement (3) is used in proving the existence of the structure even for unbounded ARGs as well as in computing the actual bound on the size. We show that the minimal structure displays the same graph-theoretic characteristic as in Statement (1) above. The interested reader may see Fig 6 in [14] for an explanation of the ‘Ancestor without Ancestry’ paradox and an illustration of its consequence in Fig 3 of the same paper.

**Figure 6:**
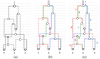
**(a) The embedded trees (b) The *sg*(*v*) at each node** Viewing the transmission of the genetic material in an *G* where the number of non-mixing segments *M =* 3. (b) An alternative view of (a) explicitly showing the flow of the ancestral material. At each node the nonmixing segment corresponding to the embedded tree is shown in the same color as that of the tree.

**Figure 3:**
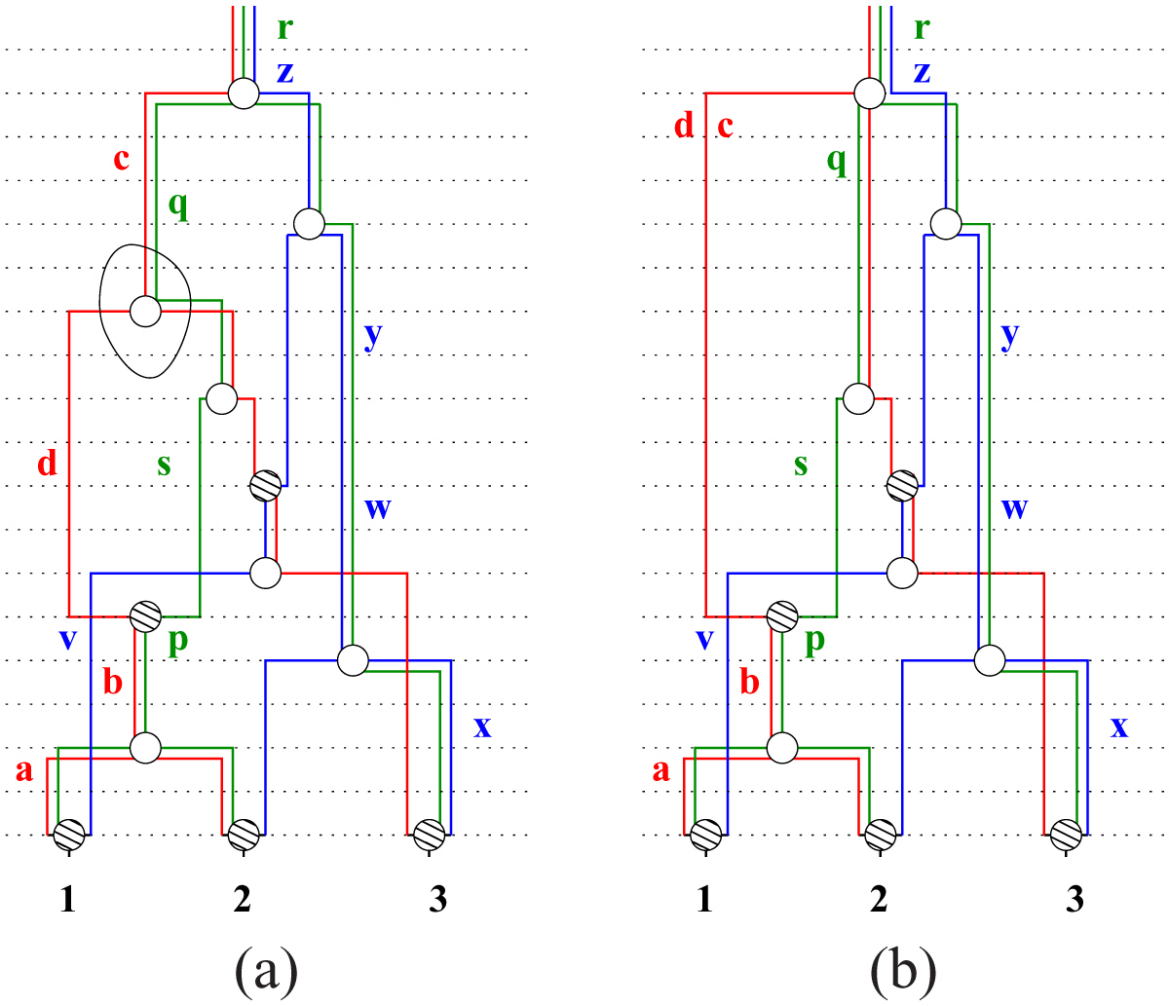
**(a) *G* (b) *G* (c) *G_md_*** Overall picture: (a) A generic ARG and all its genetic flow in (b), thus defining the samples *S*(*G*). The two marked nodes are not t-coalescent. (c) A minimal descriptor, *G_md_* as it preserves the structure of *G*. The marked nodes are t-coalescent but non-resolvable. Note that although the graphs are clearly topologically very different, yet they define exactly the same samples i.e., *S*(*G*) *= S*(*G_md_*). Note that *G_md_* preserves the structure of *G*.

## Methods

### Basic definitions

**Figure 1:**
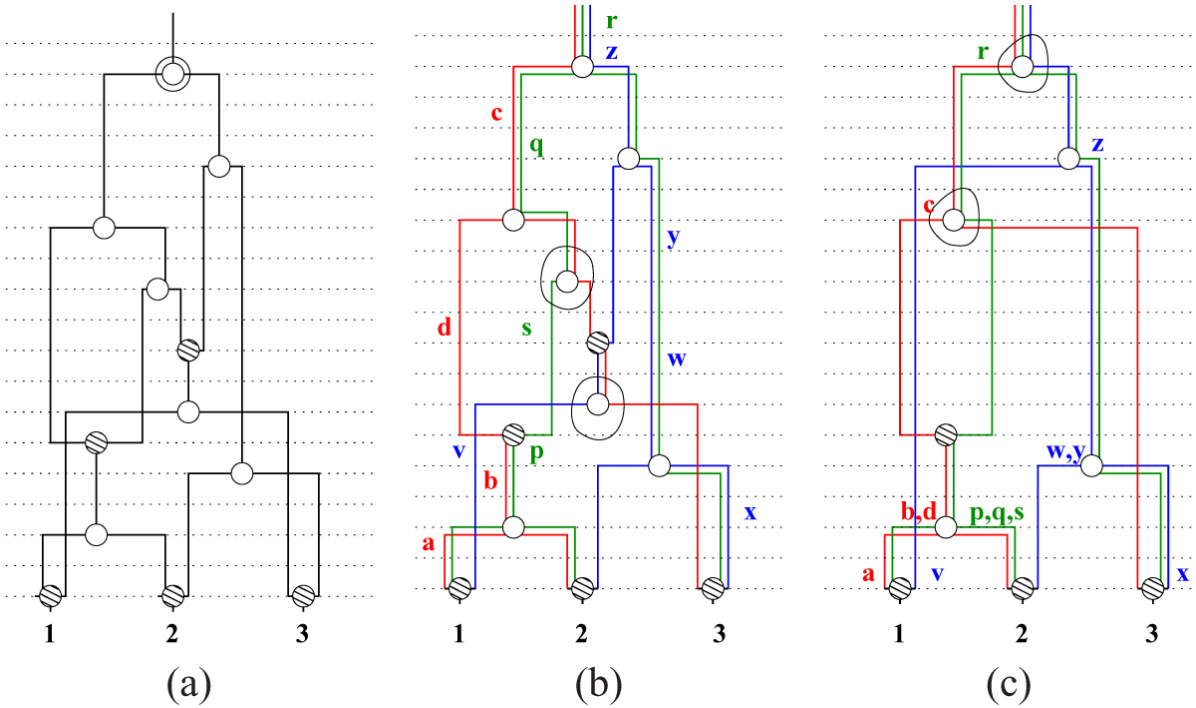
**(a) *G* (b) Three embedded trees (c) Genetic flow** The dotted horizontal lines represent time (in generation) and the extant samples, numbered 1, 2, 3, are at the bottom row. Thus *K* = 3, in this example. The genetic material flows from the nodes at a higher level to the nodes at the lower level in the figures. The hatched nodes are the genetic exchange nodes. (a) The topology of an instance of an ARG, where the GMRCA is marked by an additional ring. (b) A possible embedding of (a) by 3 trees (shown in red, green and blue respectively) by Thm 1 (2).(c) Genetic event labels on the ARG.

The ARG is usually parameterized by three essential parameters: *K* the number of extant samples, 2*N* the population size and recombination rate *r* (see texts such as [3] for a detailed description). The alternative definition suggested by Thm 1 (2) is illustrated in Fig 1(a). Here an ARG, defined on three (*K*) extant samples, is decomposed into three (*M*) trees. Note that *M* is the number of non-mixing or completely-linked segments in the extant samples. In both the models all the samples are of same length say *s* and additionally the length of each of the *M* segments is specified as *s*_1_*, s*_2_*, .., s_M_* with , in the former.

#### The graph description

For ease of exposition in the following paragraphs, let the edges be directed, the direction towards the more recent generation (or the leaves). In other words, the leaf (extant) nodes have no outgoing edges and the root node has no incoming edges. The edges of the ARG are annotated with genetic event and the labels are displayed in the illustrations. See Fig 1(c) for an example. An edge in *G* is defined to have multiple *strands*. In the illustrations, the multiple strands are shown as distinct colors, each color corresponding to one of the component trees 1 *≤ i ≤ M*. Between any pair of vertices *v*_1_ and *v*_2_, no two strands can be of the same color. Thus the number of multiple strands, corresponding the edge, between a pair of vertices can be no more than *M*.

**Figure 2:**
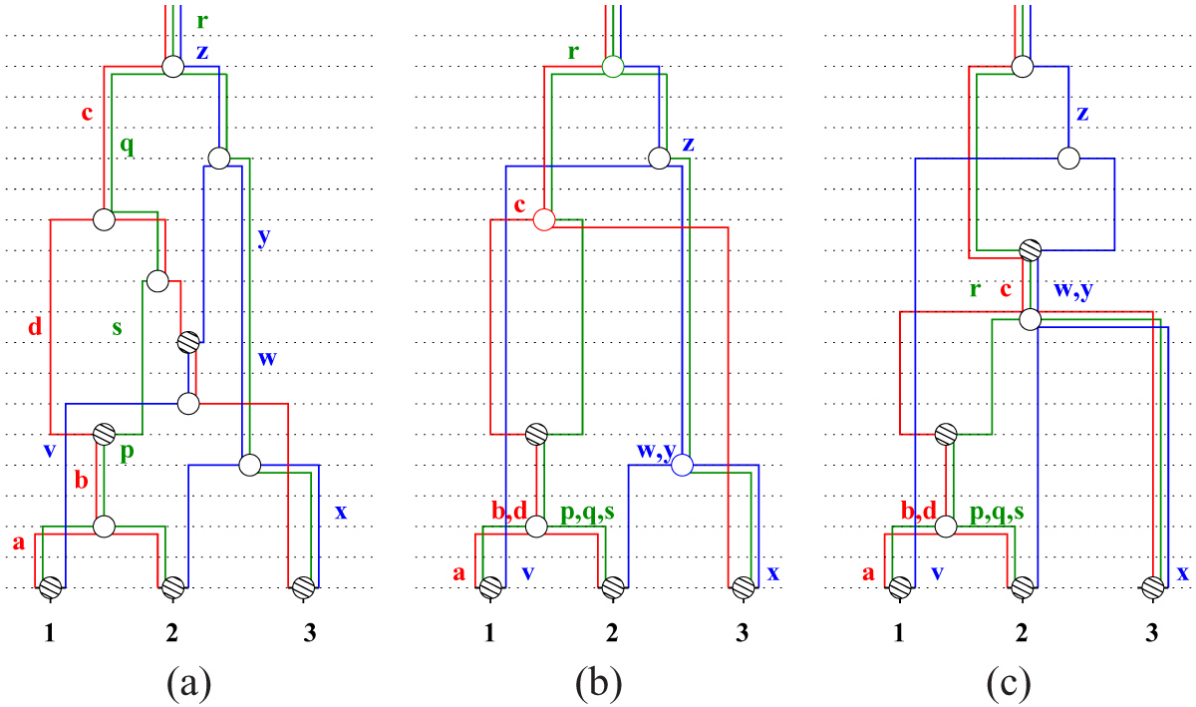
**(a) *G* with marked *v* (b) *G \* {*v*}** An example to show how *G \* {*v*} is computed. *v* is marked on *G* in (a). Note that *v* has two red and one green outgoing edges; it has one red and one green incoming edge. When *v* is removed, one of the new red strands is labeled by the set {*d, c*} and the new green strand is labeled by the singleton *q*. The resulting edges and labels are shown in (b). [Note that vertex *v* is a resolvable node in *G*.]

The annotations on the edges play a critical role since it is these annotations that ultimately shape the extant samples. In the paper, samples refer to extant samples. The two kinds of genetic events represented in the graph are (1) duplication events and (2) genetic exchange events. While the latter is modeled by the genetic exchange nodes, the former is modeled by labels on the edges. To keep this discussion simple, let the duplication genetic event correspond to Single Nucleotide Polymorphisms (SNPs). For example in Fig 1(c), the SNPs on the red tree are shown as *a, b, c, d* in red. Also, the exact position of the SNP on the genome does not matter. However, in the ARG, a particular ordering of the *M* trees is assumed and hence the SNPs of each of the *M* trees respect this order (this is reflected in the sample definitions below where red is the leftmost segment and blue the rightmost). Each strand of an edge is labeled by a set of genetic events (SNPs), possibly empty. A node with multiple ascendants (parents) is called a *genetic-exchange* node. A node with multiple descendants (children) is a *coalescent* node. Note that a node can be both a coalescent as well as a genetic-exchange node. In Figures 1, 2, 3, 4, a genetic-exchange node is displayed as a hatched disc. To summarize,

**Property 1 (ARG properties)***1. An ARG G must satisfy the following*

(a) (topology) Every node v in G must have multiple children or multiple parents (since chains are not informative).

(b) (annotations) The duplication genetic event label (say, SNP) corresponding to a position on the samples must transmit down to at least one extant sample.

*2. Further, a nontrivial G must encode at least M –* 1 *genetic exchange events.*

**Figure 3:**
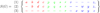
**(a) *G* (b) *G* (c) *G_md_*** Overall picture: (a) A generic ARG and all its genetic flow in (b), thus defining the samples *S*(*G*). The two marked nodes are not t-coalescent. (c) A minimal descriptor, *G_md_* as it preserves the structure of *G*. The marked nodes are t-coalescent but non-resolvable. Note that although the graphs are clearly topologically very different, yet they define exactly the same samples i.e., *S*(*G*) *= S*(*G_md_*). Note that *G_md_* preserves the structure of *G*.

**Figure 4:**
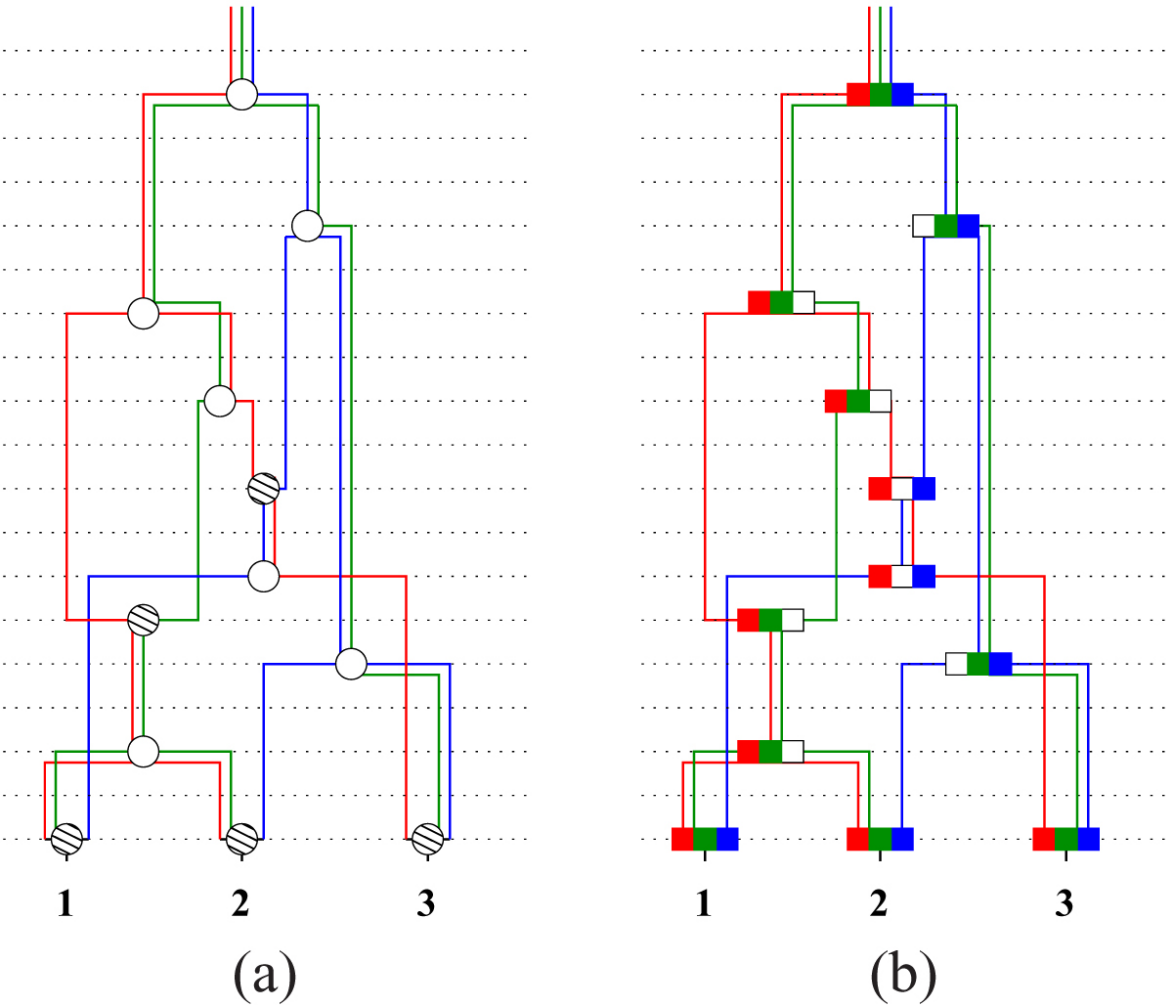
**(a) *G* (b) *G_md_* (c) ** Multiple minimal descriptors: Both *G_md_* and  are minimal descriptors of G (both samples-preservisng and structure-preserving). The two have exactly the same number of nodes, though  has more genetic exchange nodes.

#### Samples S(G)

**Figure 5:**
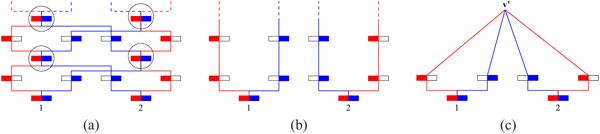
amples defined by *G* of Fig 1(c).

Next, we define the samples represented by the graph instance *G* of the ARG. This is denoted as *S*(*G*) which is a set of *K* sequences which is also the number of leaf nodes in *G*. Each sequence is obtained simply by “flowing” the genetic event labels of tree *i*, 1 *≤ i ≤ M*, along paths of color *i* all the way down to the leaf (samples) units. In the figures in the paper, we assign colors to the labels to associate the genetic events with the specific colored strand. For example, the samples defined by *G* in Fig 1(c) are shown in Fig 5. Here we have aligned and numbered the three samples as (1), (2) and (3) corresponding to the labeled leaf nodes of Fig 1(c).

**Definition 1 (samples-preserving)***G and G*′ *are samples-preserving if and only if S*(*G*) = *S*(*G*′).

When two instances are samples-preserving, all the allele statistics, including allele frequencies, LD decay and so on are identical in the two.

#### 1-vertex compactification

By Property 1 (1a), *G* should not contains chains. Consider a case where the underlying evolutionary history of *G* is unbounded. Then *G* has no GMRCA. Then it is possible that *G* contains chains of infinite length. We introduce a natural one-vertex compactification to obtain a well-defined graph *G*′ from *G* as follows. If the structure has *l* disjoint chains of infinite lengths starting from *v*_1_*,v_2_,..,v_l_,we* introduce a new vertex *v*′ and replace each infinite chain starting from node *v_j_, 1≤j≤l,* with an edge from *v*′ to *v_j_* and the label of these new edges is the union of all the component edges of the respective chains. Since the chains are disjoint, by the construction,

1. *v*′ the LCA of *v*_1_*,v*_2_*, ..,v_l_*, and

2. *G* and *G*′ are samples-preserving, i.e., *S*(*G*) *= S*(*G*′).

##### Resolvability of nodes and ARGs

To maintain biological relevance, a “missing” node is modeled by the following vertex removal operation.

**Definition 2***(G \* {*v*}) *Given G and a node v in G,G\ {v} is obtained in the following steps.*

1. For each child v_c,i_ of v, that is in the embedded tree 1 ≤ i ≤ M,

*(a) (adding new edges) this child is connected by a new edge to**the parent of v in i,*

*(b) (annotating the new edges) the new edges between**and v_c,i_ have the same label as that of the corresponding old edges between v and v_c,i_, except for exactly one such new edge whose label is the union of the labels on the two edges, i.e., the outgoing edge from v to v_c,i_ and the incoming edge from**to v.*

(This is to avoid introducing parallel mutations, i..e, the same label appearing multiple times on the embedded tree *i.)*

2. The node v with all the edges incident on it are removed from G.

See Fig 2 for an illustrative example. Notice that for the red tree, the new annotation of {*d, c*} is obtained by taking the union of the sets on the incoming and outgoing red edges on *v*, for only one (out of the two) new edges.

**Definition 3 (resolvable node)***Node v of G is called* non-resolvable *if S*(*G*) *= S*(*G\*{*v*})*. Similarly node v is called* resolvable *if S*(*G*) *≠ S*(*G \* {*v*})*.*

Next, we extend the definition of removing multiple vertices from *G*. The following is straightforward to verify and we omit the details.

**Observation 1***Given vertices v*_1_*and v_2_ in G,*

*S*((*G \* {*v*_1_}) *\* {*v_2_*}) *= S*((*G\* {*v_2_*}) *\* {*v*_1_}).

### A minimal descriptor

Next we identify the vertices in *G* that determine the topology (as well as the branch lengths) in the *M* embedded trees.

**Definition 4 (t-coalescent, structure-preserving)***(1) A coalescent vertex in G is t-coalescent if and only if it is also a coalescent node in at least one of the M embedded trees. (2) Given G and G*′*, if each of the M embedded trees in G and G*′* are identical in topology as well as branch lengths (in generations), then G*′* preserves the structure of G and vice versa.*

For example, the marked vertex in Fig 2(a) is *t-coalescent* since it is also a coalescent node of the red embedded tree.

**Theorem 2***1. A resolvable coalescent node v is also t-coalescent in G.*

*2. If G*′*←G\U and no t-coalescent vertex of G is in U, then G*′* is structure-preserving.*

*Proof* (1) Since *v* is resolvable *S*(*G*) *≠ S*(*G \* {*v*}). The number of samples corresponds to the number of leaf nodes in *G* and since *v* is not a leaf node, this number of elements in both *S*(*G*) and *S*(*G \* {*v*}) is unchanged and *|S*(*G*)*| = |S*(*G\*{*v*})*|* must hold. Since both the sets have the same cardinality, the description of the sample(s) must be different. In other words, the flow of the genetic material must be affected. Note that the genetic event annotation (say SNPs) on the edges flows to the reachable samples. Assume the contrary, i.e., *v* is not a coalescent node of any of the embedded trees. Note that the union of labels (in Step 1(b) of removing a vertex operation) on the incoming edges and outgoing edges on *v* does not affect the set of samples that carry these SNPs. However, the union of the labels of the two outgoing edges of *v* causes the samples to be different (unless the two edges have exactly the same reachable samples). This is possible only when these two outgoing edges on *v* are part of the same component, say 1 *≤ i ≤ M*, and thus *v* is a coalescent node of the component tree *i*. This contradicts the assumption, proving Statement (1).

(2) The topology and the individual branch lengths of each tree *i*, 1 *≤ i ≤ M*, is defined completely by the coalescent nodes of the tree *i*. Since *U* does not contain any of the coalescent nodes of any of the *M* trees, then the structure of *G*′ is preserved by Definition 4.

The theorem shows that the vertices that ensure the invariance of the branch lengths of each embedded tree are also resolvable, leading to the following.

**Definition 5 (minimal descriptor)***(1) An ARG G is a minimal descriptor if and only if every coalescent vertex, except the GMRCA, is t-coalescent. (2) An ARG G_md_ is a minimal descriptor of G if and only if (a) G_md_ preserves the structure of G, (b) G_md_ is a minimal descriptor, and (c) G and G_md_ are samples-preserving, i.e., S*(*G*) *= S*(*G_md_*) *holds*.

The following gives a constructive description of a minimal descriptor.

**Observation 2***Given G, let U be the set of all coalescent vertices in G, other than the GMRCA, that is not t-coalescent. Let G*′* ←G\U. Then G*′* is biologically and evolutionary relevant as*

*1. (samples-preserving) the allele statistics (including allele frequencies, LD decay) in the samples in both G and G*′* are identical, and*

*2. (structure-preserving) the embedded (marginal) trees of G and G*′* are identical.*

*In other words, G*′* is a minimal descriptor of G.*

Proof: By Definition 5 (1) and the following (which can be verified), *G*′ is a minimal descriptor.

*Let v_1_ be a t-coalescent vertex and v_2_ a non t-coalescent vertex in G. Then v_1_ continues to be a t-coalescent vertex in G\*{*v_2_*}*. Further if V*_1_*is a set of t-coalescent vertices, and V_2_ a set of non t-coalescent vertices in G, then each v* ∈ *V*_1_*continues to be t-coalescent in G\V_2_.*

(1) It follows from Thm 2(1) that if *v* is not t-coalescent then *v* is not resolvable, hence by Obs 1, *S*(*G*) *= S*(*G*′). Then it follows that the allele statistics must be identical since the samples are. (2) It follows from Thm 2 (2) that *G*′ is structure preserving. Hence the topology, as well as the branch lengths, of each *M* embedded tree is the same in *G* and *G*′. Thus, by Definition 5 (2), *G*′ is a minimal descriptor of *G*.

#### Flexibilities of ARG structures

Fig 4 shows two distinct minimal descriptors for an ARG *G*. This is partly due to the flexibility of a network topology, in contrast to say a tree topology. For instance, we demonstrate here how seemingly unrelated nodes can be potentially merged into one with neither affecting the samples nor the embedded tree structures. This can be systematized as follows, although the merge operation, defined below, is not biologically interpretable in an obvious way.

**Observation 3***Let v*_1_*and v*_2_*be two vertices in an ARG G such that (1) there is no directed path from v*_1_*to v*_2_*or v*_2_*to v*_1_*and* (*2*) *sg*(*v*_1_) ∩ *sg*(*v*_2_) = ø. *We define a* node-merge *operation of v*_1_*and v*_2_*as follows. ARG G*′* is obtained from G by removing v*_1_*and v*_2_*and introducing a new vertex v*_3_*where the incoming edges of v*_3_*is the union of that of v*_1_*and v*_2_*and similarly the outgoing edges of v*_3_*. Then the node-merge operation is structure-preserving and samples-preserving (S*(*G*) = *S*(*G*′)).

The first condition ensures that the merging does not introduce directed cycles in the graph thus maintaining the integrity of the ARG structure. The second condition ensures that the two vertices affect non-overlapping portions of the sample space. Hence merging the two nodes neither affects *S*(*G*) nor the embedded trees.

In fact it is even possible to merge more than two vertices and additionally, under more relaxed constraints than in Obs 3. The example in Fig 4(c) shows a “merging” of three t-coalescent nodes of (b) (shown as red, blue and green hollow circles in (b)).

## Results

### Properties of minimal descriptor ARGs

Given the topology of an ARG *G* with the embedded (marginal) trees and annotations representing genetic events, we have seen that this defines an unambiguous genetic flow giving rise to the samples *S*(*G*). This annotation also implicity defines the segments associated with each node. The *M* embedded trees in *G* correspond to *M* segments on the chromosome of the *K* samples which is encoded by the leaf nodes of *G*. We assume that these *M* segments of interest are consecutive on the chromosomes of the samples. Thus these trees can be numbered by consecutive integers from 1 to *M*, the values indicating the order on the chromosomes. Thus the multiple edges of *G* (defined in the last section as colored edges) implies an annotation of a node *v* as well. We define this as *sg*(*v*) which is formalized below.

**Definition 6 (*sg(v)* overlap)***Given node v in an ARG G, sg*(*v*) *is the set of the embedded trees that v is incident on. Two vertices v*_1_*and v*_2_*in G are said to overlap if sg*(*v*_1_) ∩ *sg*(*v*_2_) ≠ ø.

In [14], this is defined as *gm*(*v*), however in this paper we use *sg*(*.*) to avoid confusion with the SNPs as genetic events. For example, consider the marked node *v* of Fig 2(a). Let the red, green and blue trees be numbered 1, 2 and 3 respecting the order in which they appear on the chromosomal segment as defined in the samples in Fig 5. Then *sg*(*v*) *=* {1, 2}. Also, let the leaf nodes marked 1, 2 and 3 of the same tree in Fig 6(a) be *u*_1_*,u_2_,u_3_* respectively. Then

*sg*( *u*_1_) *= sg*(*u_2_*) *= sg*(*u_3_*) *=* {1, 2, 3}.

Fig 6(b) displays these segments at each node. To simplify the exposition, assume that the *M* segments are numbered consecutively from 1 to *M*.

**Definition 7 (gapped/ non-gapped node)***A node v in ARG G is called a gapped node if the elements of sg*(*v*) *are not consecutive. A non-gapped node is a node that is not a gapped node.*

Note that when the elements of *sg*(*v*) are not consecutive, or *v* is gapped, it implies that while tracking the relevant chromosomal segments in the ARG, *v* appears to be carrying segments that matter (i.e., are ancestral to the corresponding segments of some of the samples) and are interspersed with segments that do not matter (i.e., are not ancestral to the corresponding segments in any of the samples).

#### On boundedness of ARGs

**Figure 7:**
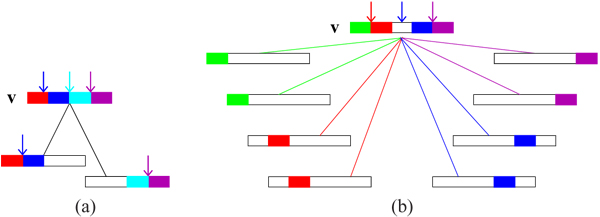
**(a) Unbounded *G* (b) *G*′*←G\U* (c) *G_md_*** Bounded *G_md_* of unbounded *G*: (a) An unbounded *G*. Here *K* = 2 corresponding to the samples numbered 1 and 2 and *M* = 2, for the two segments colored red and blue. The pattern of vertices and edges can be repeated along the dashed edges to give an unbounded structure. The coalescent nodes that are not t-coalescent are marked by circles: let *U* be the set of all such nodes. (b) *G*′ is obtained from *G* after removing the vertices in *U*. (c) *G_md_* is obtained by the 1-vertex compactification of *G*′ with the new vertex *v*′.

A generic ARG is not necessarily bounded, i.e, it can have an infinite number of vertices. See Fig 7(a) for an example of such an ARG.

**Theorem 3***An unbounded ARG G always has a bounded minimal descriptor.*

*Proof:* We prove this by constructing a bounded minimal descriptor from the unbounded *G*. Since *G* is unbounded, *G* has no GMRCA. Obtain *G*′ from *G* by removing all coalescent vertices that are not t-coalescent. Then by definition *G*′ is structure-preserving and *S(G) = S(G*′).

By Thm 1 (2), *G*′ has *M* embedded trees. For each embedded tree *T_i_* , let *L_i_* be the set of LCAs in *G*. Note that the LCA of a tree is completely defined topologically. Let . Then we prove the following:

*The only infinite chains in G*′* originate in a vertex in L and they are disjoint.*

First we need to show that any infinite chain in *G*′ must originate in a vertex in *L*. Assume the contrary that there is a chain originating in *v* ∉ *L*. Let *u* be an extant sample reachable from *v* and let *i* ∈ *sg*(*u*), then *v* must be an LCA in tree *i* and then *v* ∈ *L* by construction, which is a contradiction. Next we need to show that no two chains overlap. Assume the contrary that a pair of infinite chains, one originating in *v_1_* ∈ *L* and the other in *v_2_* ∈ *L*, cross paths, say at *u*. Since it is a chain, *u* is a coalescent node and not the GMRCA. If *sg*( *v*_1_) ∩ *sg*(*v_2_*) *=* ø, then it contradicts the fact all (non-GMRCA) coalescent nodes in *G*′ must be t-coalescent. But if *i* ∈ *sg*( *v*_1_) ∩ *sg*(*v_2_*), then it contradicts the fact that *v_1_* and *v_2_* are LCAs of the embedded tree *i*. Hence no two chains can cross.

(B) and (C) prove the statement. Then since the preconditions hold, we can apply the 1-vertex compactification to *G*′ by adding the new vertex *v*′ and obtain *G*″. To show that *G*″ is a minimal descriptor of *G* using Definition 5, we next assert the following three statements:

*(i) S(G″) = S(G), (ii) G*″* preserves the structure of G and (iii) v*′* is the GMRCA of G*″.

From (A) and the definition of the 1-vertex compactification process, statements (i) and (ii) hold. Also by the construction, *v*′ is the LCA of all the vertices in *L*. Hence by Thm 1 (3), *v*′ is the GMRCA of *G*″. This concludes that *G*″ is a minimal descriptor of *G* with the GMRCA *v*′, hence bounded.

**Corollary 1***The degree of the GMRCA in a minimal descriptor is ≤ MK.*

This follows from the cardinality of *C* in the proof. Since each segment can have no more than *K* LCAs, *|L| ≤ KM*.

Fig 7 illustrates the construction procedure used in the proof of the theorem on a simple example with *K* = *M* = 2. The corollary states that even if the underlying ARG *G* is unbounded, there exists a minimal descriptor with a GMRCA with not just finite but *a priori* bounded degree.

#### Back to properties of ARGs

The previous section gave a global characteristic of a minimal descriptor ARG in terms of resolvable nodes. In this section we explore properties of nodes of the minimal descriptor ARG that can be determined by studying a very local neighborhood of the node.

**Theorem 4***1. If a coalescent node v, except the GMRCA, in a minimal descriptor ARG has descendants u*_1_*, u*_2_*, .., u_l_, for some l >* 1*, then for any u_i_, there exists u_j_, i ≠ j, such that*

*sg*(*u_i_*) ∩ *sg*(*u_j_*) *≠* ø.

*In other words, for each descendant u_i_ of v there exists another descendant u_j_ of v overlapping with u_i_,1 ≤ i ≠ j ≤ l*.

2. The number of vertices in a minimal descriptor ARG is finite. Moreover, if n_c_ the number of coalescent events, n_e_ is the number of genetic exchange events, and n_v_ the number of vertices in a nontrivial minimal descriptor ARG, excluding the leaf nodes, then the following holds:

1 *≤ n_c_ ≤ M*(*K –* 1) *+* 1, (1)

0 *≤ n_e_ ≤ K*(*M –* 1) *+ M*(*K –* 1), (2)

*n_v_* = *O*(*MK*). (3)

*Proof:* (1) Since, by Definition 5, every coalescent node in the minimal descriptor is t-coalescent, this must hold. (2) By Thm 3, every minimal descriptor ARG has a GMRCA. Also the GMRCA is a coalescent node. In *G* there are *kM* segments that eventually coalesce into *M* segments. The smallest number of coalescences occurs when all vertices coalesce into a single vertex, the GMRCA, giving a lower bound of 1. Again, by (1), since every coalescent vertex must be the coalescence in at least one embedded tree, the number of coalescent vertices, excluding the GMRCA, in a minimal descriptor ARG is no more than (*K –* 1)*M*. By Thm 1 (3), including the GMRCA introduces only one more node and this proves *n_c_ ≤ M*(*K –* 1) + 1 of Eqn 1.

**Figure 8:**
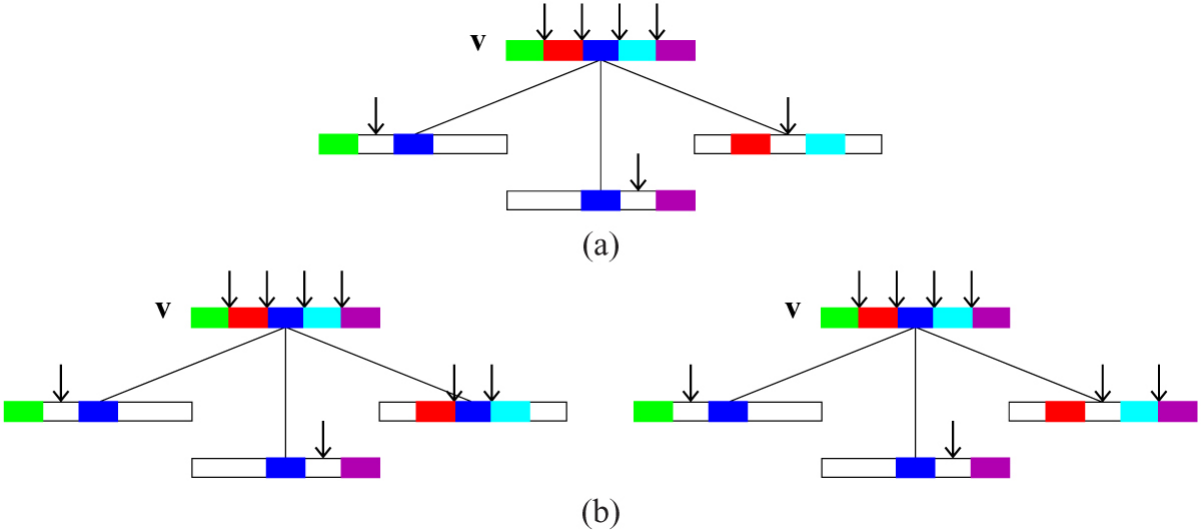
**(a) Non-resolvable node *v* (b) t-coalescent node *v*** The arrows point to the junctions in the segment. In each of the two there is an increase in the number of junctions from the descendants to the coalescing vertex *v*. (a) A non-resolvable node and the coalescence of two descendants has produced an additional junction, shown by a cyan arrow. (b) A t-coalescent node and the coalescence of the eight descendants. The four coalescences give rise to three junctions.

By the definition of the parameters of the ARG, *G* must have at least one genetic exchange vertex encoding (*M –* 1) genetic exchange events. However, the above count excludes the leaf nodes and it is possible to encode these (*M –* 1) events in a single genetic exchange leaf node. Hence a lower bound of 0 for *n_e_*. When a vertex in the ARG is displayed as a linear ordering of the non-mixing segments with distinct colors for each segment (as in Fig 6(b)), then the potential *junctions* for the exchange events are at the junction of the colored segments. Recall that by the definition of the ARG, each unit has at most *M* non-mixing segments, hence can have no more than *M –* 1 genetic exchange events. Thus there are *K*(*M –* 1) such junctions potentially each representing a recombination (or exchange) event. We adopt the following convention: each non-mixing segment in a vertex *v* contributes to a junction to its left. Thus, by this convention, the left-most segment has no associated junction. See vertex *v* in Fig 8 as an illustration. Each distinct non-mixing segment is shown by a distinct color; gap is shown as a white segment and junctions are marked by arrows of the same color as that of the segment associated with it. Thus the green, red, blue, magenta colored segments show the associated junctions, and the leftmost green segment has none in Fig 8(b). We call the non leftmost as interior segments. Also, note that a gap does not contribute to a potential junction by our convention. For a recombination event to occur multiple times at the same junction involving the same or similar set of samples, it is clear that a coalescence must occur. Then the following can be verified.

Each coalescent event of an embedded (marginal) tree can create at most one additional junction in the coalescing node.

**Figure 9:**
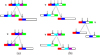
**(a) Non-resolvable node *v* (b) Two distinct configurations for t-coalescent vertex *v*** Examples to illustrate the effect of overlap of descendants of vertex *v* on the number if junctions in *v*. (a) The rightmost descendant does not overlap with any of the two left descendants. In this case the number of junctions increase by one. (b) The three descendants, in each of the two cases, satisfy the conditions of Thm 4 (1) and there is no increase in the number of junctions.

**Figure 10:**
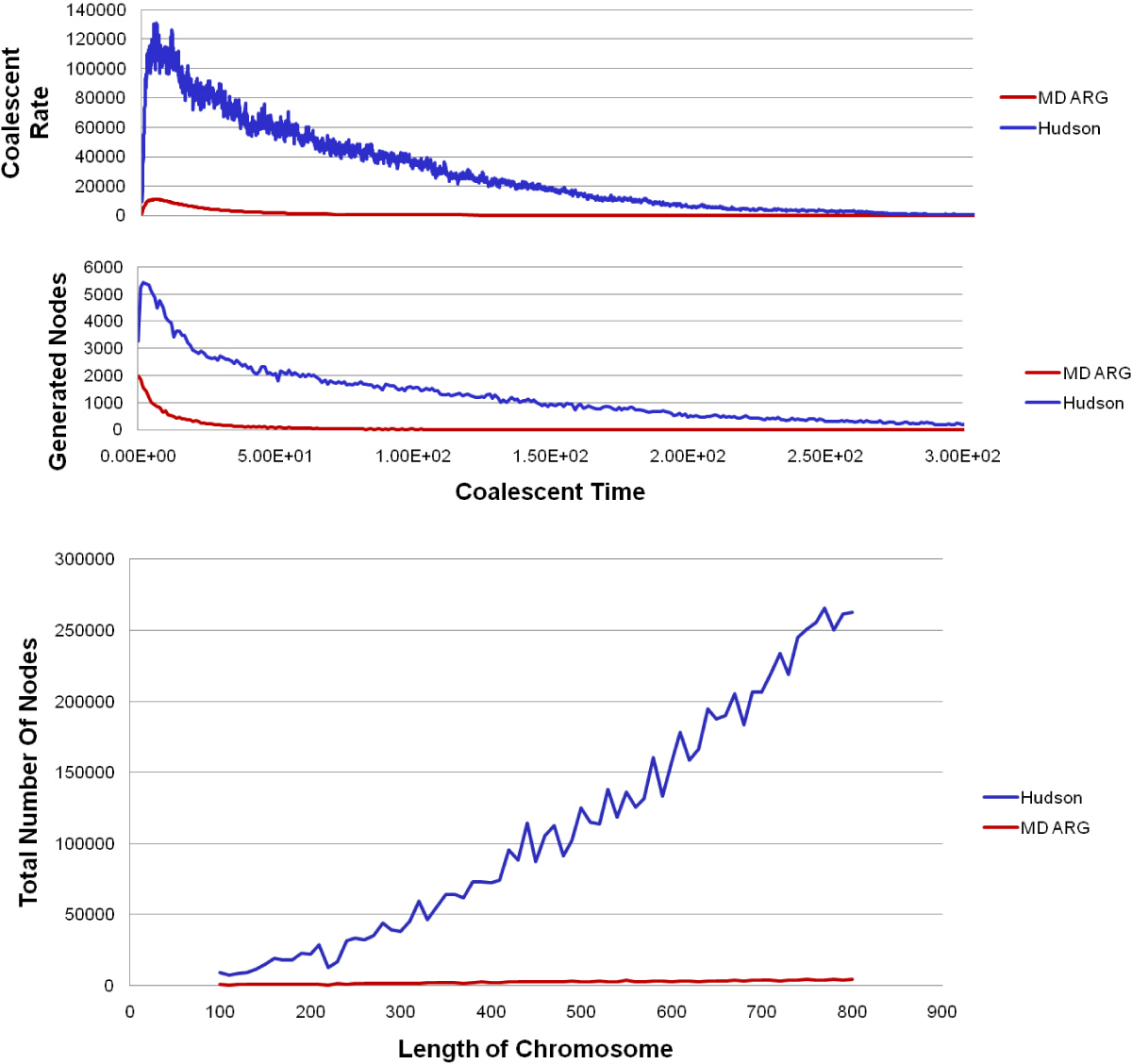
**(a) Non-gapped segments (b) Gapped segments** t-coalescent vertex *v*: Five cases are shown where the total number of junctions does not increase with coalescence. The white segments represent the gaps whereas the colored segments refer to the non-mixing segments. (a) Two cases with non-gapped segments. (b) Three cases with gapped segments.

Consider Fig 8. In (a), *v* is not t-coalescent. In (b) four coalescences in four embedded trees produces three junctions in the coalescing node. Since the total number of coalescent events in the embedded trees is no more than *M*(*K –* 1), the additional junctions is bounded by the same number. Hence *n_e_ ≤ K*(*M –* 1) + *M*(*K –* 1) of Eqn 2. See Figs 9-10 for illustration of other scenarios where there is *no* increase in the number of junctions in the coalescing vertices. The coalescent events are absorbed in the worst case scenario in the count of genetic exchange events. Next, Eqn 3 follows from Eqns 1 and 2.

**Corollary 2***The minimal descriptor satisfies the bounded-degree property (of Thm 1 (1)).*

By Corollary 1, there is an *a priori* bound on the GMRCA. By the theorem there is such a bound on all the other vertices. Hence the result.

### Binary ARGs

The sampling algorithms incorporating the coalescent process produces ARGs that bound the indegree and outdegree of a node to two [3]. We call such ARGs as binary ARGs and they also give stronger characteristics that can be further exploited by the sampling algorithms. We find that, to prove these characteristics, it is not necessary to restrict the incoming edges of a node to two.

**Definition 8 (binary ARG)***A vertex in a binary G has no more than two outgoing edges, except the GMRCA.*

We next identify some properties on these binary ARGs that can be again used in the sampling algorithms, if required. Consider the scenario where the genetic exchange event is restricted to recombinations. In other words if a node *v* has two incoming edges them the genetic material of *v* is split such that the left part of the segment is derived from one of the parental nodes and the right part is derived from the other parent. This is in contrast to an arbitrary segment being derived from one and the remaining from the other parental node. Next, we prove a rather unexpected property of a node of a binary minimal descriptor ARG under recombinations: the genetic material carried by a node has no gaps. This result is somewhat counter-intuitive (since it appears unduly restrictive and is counter to earlier beliefs) and is proved in (2) below.

**Theorem 5***1. If n_c_ is the number of coalescent events, n_e_ the number of genetic exchange events, and n_v_ the number of vertices in a nontrivial binary minimal descriptor, excluding the leaf nodes, then the following holds:*

(*K –* 1) *≤ n_c_ ≤ M*(*K –* 1) *+* 1, (4)

0 *≤ n_e_ ≤ K*(*M –* 1), (5)

*n_v_* = *O*(*MK*). (6)

2. Let (a) the genetic exchange events be restricted to recombinations and (b) all the leaf nodes be non-gapped. Then no node in a binary minimum descriptor is gapped.

*Proof* (1) The proof of Eqn 4 is along the lines of that of Eqn 1. We show *n_e_ ≤ K*(*M -* 1) of Eqn 5 as follows. By the definition of the ARG, each unit has at most *M* non-mixing segments, hence can have no more than *M –* 1 genetic exchange events. Thus there are *K*(*M –* 1) such junctions. For a recombination event to occur multiple times at the same junction involving the same or similar set of samples, it is clear that a coalescence must occur. Following the notation used in the proof of Thm 4, We next prove the following statement:

*If there is some overlap in the coalescing vertices u*_1_*,u_2_, then there is no increase in the number of junctions from the sum total in u_1_ and u_2_ to that of the coalesced vertex v.*

Every non-mixing segment of *v* corresponds to the same non-mixing segment in at least one of the descendants *u_1_,u_2_*. There can be an increase in the junctions in *v* if and only if a leftmost non-mixing segment in say *u*_1_, is not the leftmost in *v*. It cannot be a leftmost in *u_2_* as well, since it is not a leftmost in *v*. Thus it is an interior in *u_2_*. Thus this segment has a corresponding junction in *u_2_* that contributes to the junction in *v* without introducing an increase in the count of junctions. Since a coalescence does not increase the count of junctions, *n_e_ ≤ K*(*M –* 1) holds. Next, Eqn 6 follows from Eqns 4 and 5. This completes the proof of Statement (1).

(2) Assume the contrary, i.e., there exist gapped nodes in *G*. Amongst all such nodes, consider a *least* node *v*, i.e., *v* is such that there is no other gapped node in all the paths from *v* to the reachable nongapped leaf nodes. Let *v* have only one child *u*. Since the only genetic exchange event is recombination, *u* must be gapped for *v* to be gapped. This leads to a contradiction that *v* is the least such node. Then *v* must have two children. Consider the two following cases. Case i: Let the two descendant nodes of *v* be coalescent nodes. By the assumption each of them is non-gapped and by Thm 4 (1) the two must overlap. Hence *v* must be non-gapped as well. Case ii: Let at least one of the descendant nodes of *v,* say *u,* be a genetic-exchange node. Again, since the only genetic exchange event is recombination, the segment transmitted by *u* to *v* is nongapped. Hence by Thm 4 (1) *v* is nongapped. Thus for *v* to be gapped at least one of its two descendants must be gapped, leading to a contradiction.

#### Comparison with the standard coalescent

We introduce a definition of equivalence of ARG instances here.

**Definition 9 (equivalent ARG instances)***Let G and G*′* be two ARG instances with G = ∪_T∈Τ_T and G*′* = ∪_T∈Τ′_T where each T is a tree. G and G*′* are said to be equivalent if and only if the following conditions hold. (1) S(G) = S*(*G*′)*, i.e., both define the same set of samples and (2) there exists a bijection f : Τ→Τ′ such that for every T∈Τ, f*(*T*) *is isomorphic to T via an edge-length as well as leaf-label preserving isomorphism*.

A graph-theoretic isomorphism definition, for the equivalence of two ARG instances, is unduly rigid and this weaker, but more relevant, definition of equivalence is derived from the typical handling of ARGs in literature [13,17-19]. Next, we prove the following.

**Theorem 6***Given an instance of ARG G, its minimal descriptor G_md_ is equivalent to G. (In this sense, the minimal descriptor of a binary ARG is the standard coalescent model.)*

The equivalence of *G* and *G_md_* follows from Defns 5 and 9. (Further, since the binary ARG is an alternative modeling of the standard coalescent, the minimal descriptor of the binary ARG is equivalent to the standard coalescent model.)

Furthermore, in our experiments that involved comparison with other models utilizing the recombination rate parameter, *r*, we enforce this parameter on the minimal descriptor to simplify the task of comparison. We observe empirically that properties such as embedded (marginal) tree branch length distribution, LD decay of samples etc match very well with that of *MS*[15] and *GENOME*[16] although the first method uses the coalescent time to the next event and the second carries out the simulation at every generation. However, that is not the case with the approximate models [17-19].

### Estimating redundancy in an ARG

Recall that in *G* there is no vertex that has one descendant and one ascendant (Property 1 (1)). We call *G reduced* if it has no vertex that has only one descendant. Given *G,* if *G*′ is obtained from *G* by removing all and only those vertices that have single descendants then *G*′ is called as *reduced G*. Since a coalescent node cannot have a single descendant the following is easily verified: *If G*′* is a reduction of G then G*′* is structure-preserving and S*(*G*) *= S*(*G*′). A reduced *G* is a canonical form and then it is meaningful to compare the number of vertices between canonical forms.

**Observation 4***If G_md_ is a minimal descriptor of G, then the number of vertices in reduced G_md_, which is no more than in reduced G.*

We have shown that an unbounded *G* (with infinite number of vertices) always has a minimal descriptor (with number of vertices = *O*(*MK*)). Hence we focus on a subspace here- that of binary minimal descriptors. To estimate their number, we use Thm 5 (2) that states no node in a binary minimal descriptor is gapped. Characterizing the type of nodes as gapped and non-gapped, we simply compare the cardinality of the respective ‘universal sets’ of nodes of binary ARGs and binary minimal descriptors, for a rough estimate. The following is the ratio of non-gapped to gapped configurations, where *M* is the usual parameter:(7).

### Sampling algorithm

An ARG construction has the following two independent tasks: (1) Constructing the topology of the structure including the lengths (or time estimates) of the edges. (2) Annotating the edges of the structure with genetic events, the number of the events (say, mutations) is a function of the length of the edges. The topology is a critical part of the ARG and the graph-theoretic treatment of the problem isolates the topology which has lead to various novel insights. Due to the fundamental characteristics in capturing the essence of the evolving population and its versatility, the standard coalescent approach [12,15] can be used to estimate the lengths of the edges in the topology. Thus even with the graph-theoretic treatment of the problem, we appeal to essentials of coalescent theory for the sampling of the minimal descriptors. All the methods discussed in the introduction section are based on the standard coalescent model, which is analogous to the binary ARGs in this paper. Hence we focus only on this subspace of generic ARGs in this section.

Based on the models presented on this paper, there are at least two possible approaches to sampling the space: (1) One is sampling the space of standard ARGs. (2) Since a minimal descriptor is also an ARG the other approach is to directly sample the subspace of standard ARGs, corresponding to the binary minimal descriptors. In the first approach, a standard ARG is sampled and its minimal descriptor is extracted either as a post-processing step or simultaneously during the construction process. This approach has the advantage that the sampling distribution is exactly the same as that of the standard ARG, which is well studied in literature. The second approach is to directly sample a subspace of ARGs. A theoretical (time-expensive) sampling algorithm with the ‘true’ probabilities for a generic ARG is presented in [14]. Though extensively in use, the sampling distribution of the standard ARGs (binary) of the practical algorithms is not fully understood (see, for instance, discussions in [18]). Thus an understanding of the sampling distribution of a subspace of ARGs may be equally elusive.

There are two local properties of the vertices of a minimal descriptor: (1) every coalescent vertex is also a coalescent in one of the *M* embedded trees (Thm 2), and, (2) a coalescing vertex must overlap with another (Thm 4). Since these are local properties, it is possible to exploit one or both of these (related) properties in the sampling algorithms in both the above approaches. In our experiments we have used the overlap property of Thm 4 in the second approach of the last paragraph to sample the space of minimal descriptor of binary ARGs. To recapitulate, the input parameters to our sampling algorithm are: (a) *N*(*≥* 1): population size is 2*N* at each generation. (b) *K*(*>* 1): the number of samples. (c) *M*(*≥* 1) & (*s*_0_*,s*_1_*,s*_2_*,…,s_M_*): *M* is the number of nonmixing segments. The lengths of the segments are (*s*_0_*,s*_1_*,s*_2_*,…,s_M_*) that model varying recombination rates along the chromosome.

## Discussion

Based on the graph-theoretic treatment of the problem, we first identify resolvable nodes in an ARG, i.e. a node that has a possibility of being detected by *some* algorithm. A non-resolvable node has no impact on the extant samples, thus cannot be resolved without additional information. Next, we identify the structure-preserving nodes, i.e., the nodes critical to the structure of all the embedded (marginal) trees. Combining these two characteristics, we give a method-independent definition of a minimal descriptor that is both structure-preserving and samples-preserving. We prove that even an unbounded ARG has a bounded minimal descriptor. However, the ‘missing’ and ‘extra’ nodes in the model continue to be a source of unavoidable complexity.

### Missing nodes & Extra nodes

Since a minimal descriptor can be viewed as a full ARG with missing nodes, then what are these nodes that are ignored? These carry some sets of the non-mixing segments and it turns out that whether they are transmitted in a single unit or in multiple units has no bearing either on the extant samples or on the embedded (marginal) trees. This also implies that if a multitude of such nodes is injected into an ARG, it will continue to be structure-preserving with no impact on the extant samples, thus giving an inflation of the putative volume of evolutionary events. Then, their complete elimination also suggests a possible deflation. Additionally, there are a myriad ways to merge nodes (see Methods) that are both structure-preserving and samples-preserving, but alter the nature as well as the volume of the genetic events. Thus it is not immediately apparent how such intense variabilities, within the invariants (structures and samples) can be effectively tackled. It is best left to the specific question being addressed and the data at hand.

Nevertheless, due to the local properties of the nodes of the models, it may be still possible to exploit these characteristics. Recall that while the nodes in the generic minimal descriptor may be gapped, no node in a binary minimal descriptor is gapped (Thm 5 (2)). Additionally the number of nodes in the minimal descriptor of both the generic and the binary ARG is *O*(*MK*) (Thms 4 and 5).

## Conclusion

By isolating the topology of the ancestral recombinations graph, we have identified a lossless structure-preserving subgraph of the general ARGs termed the minimal descriptor ARG. We have also identified a subclass of ARGs, binary minimal descriptors, that are analogous to the *standard coalescent model* in literature. Two interesting observations are: (1) every ARG, including unbounded ARGs, has an a priori bounded minimal descriptor, and, (2) the decrease in the number of nodes from an ARG to its minimal descriptor is estimated to be exponential.

**Figure 11:**
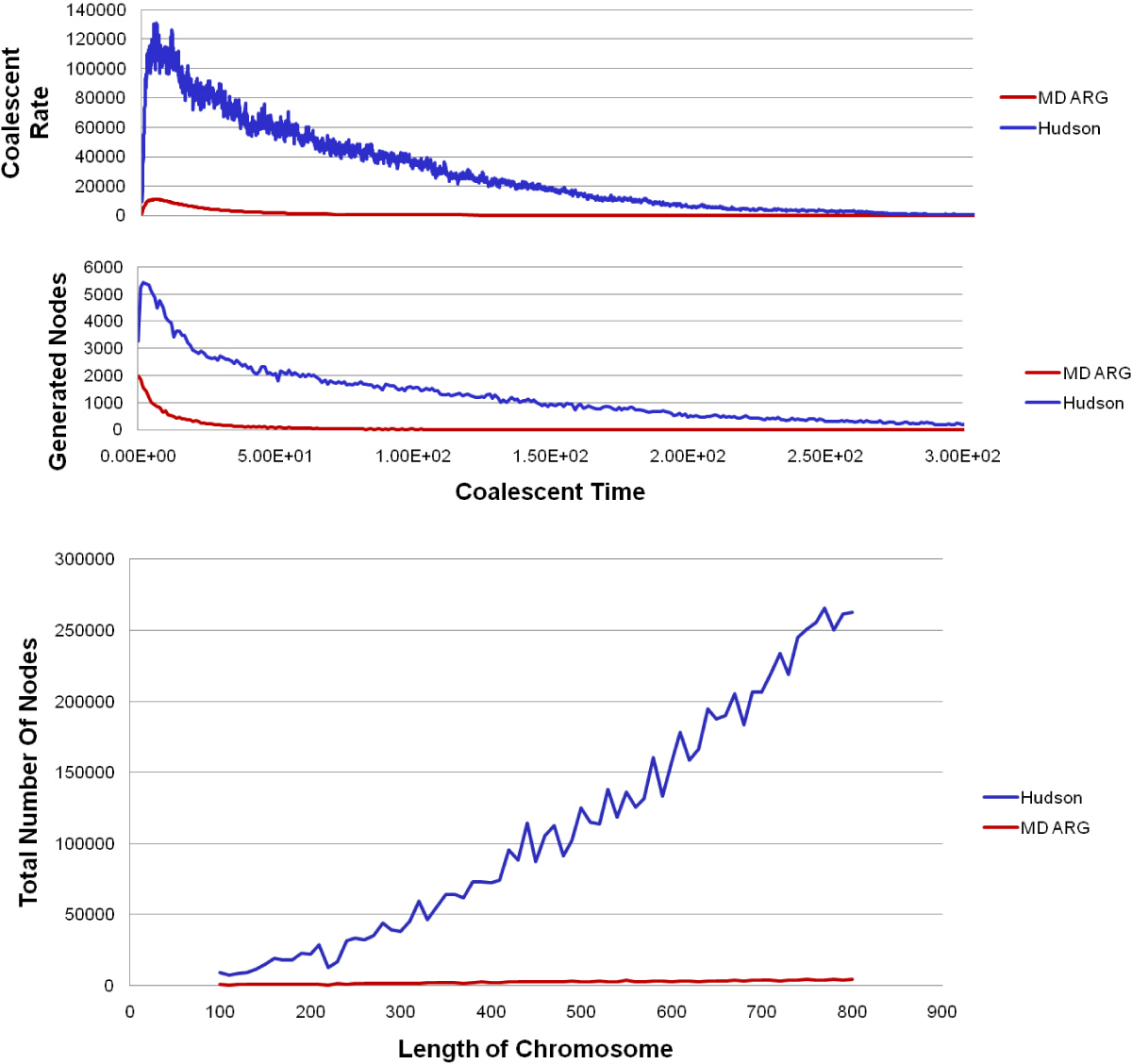
Comparison with standard ARG, MS [15], termed ‘Hudson’ in the figure, and the minimal descriptor ARG, termed ‘MD ARG’.

**Figure 12:**
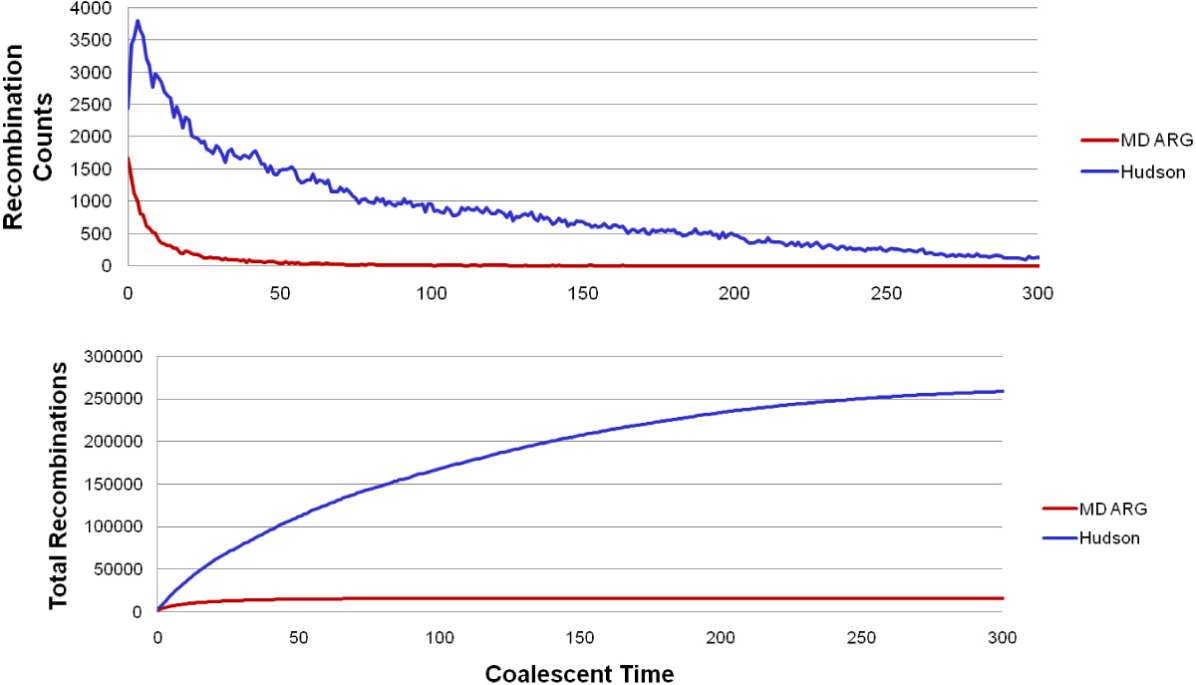
Comparison of the number of recombination nodes between standard and minimal descriptor ARG.

**Figure 13:**
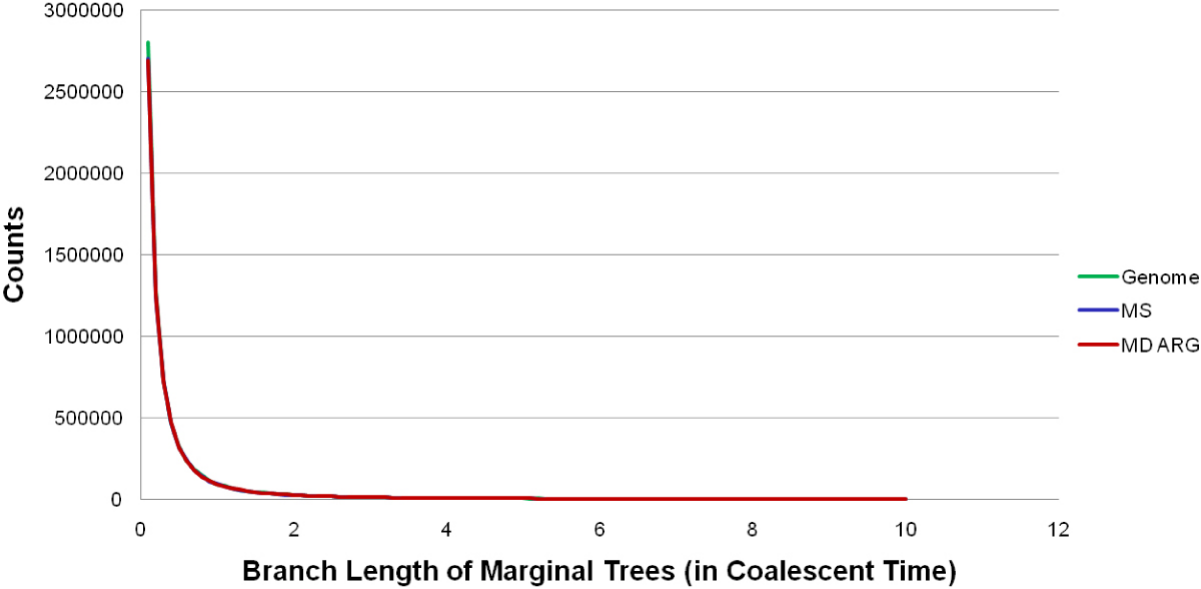
The branch length comparisons with GENOME [16] (that does not use the standard coalescent model) and MS [15]. Due to the structure-preserving property of MD ARG, the lengths match very well.

Some preliminary results from our implementation is shown in Figs 11, 12, 13 for the interested reader.

## Competing Interests

The authors declare that they have no competing interests.

## Authors contributions

LP conceived and designed the work. PFP and AJ carried out the experiments and contributed ideas.
